# Supplementary material for: Oxygenic photosynthetic responses of cyanobacteria exposed under an M-dwarf starlight simulator: Implications for exoplanet’s habitability
Source: Front Plant Sci. 2023 Feb 7;14:1070359. doi: 10.3389/fpls.2023.1070359 (PMC9941696; doi:10.3389/fpls.2023.1070359)
Supplement: Supplementary file 1 [file DataSheet_1.docx]

**Supplementary Material**

**Oxygenic photosynthetic responses of cyanobacteria exposed under an M-dwarf starlight simulator: implications for exoplanet's habitability**

Battistuzzi M.^1,2*^, Cocola L.^3^, Claudi R.^4^, Pozzer A.C.^1,4^, Segalla A.^1^, Simionato D.^1ǂ^, Morosinotto T.^1,2^, Poletto L.^3^, La Rocca N.^1,2*^

^1^ Department of Biology, University of Padua, Padua, Italy

^2^ ”Giuseppe Colombo” Center for space studies and activities (CISAS), University of Padua, Padua, Italy

^3^ National Council of Research of Italy, Institute for Photonics and Nanotechnologies (CNR-IFN), Padua, Italy

^4^ National Institute for Astrophysics, Astronomical observatory of Padua (INAF-OAPD), Padua, Italy

^ǂ^ present address: Croda Italiana spa, Altavilla Vicentina, Italy

*** Correspondence:**Corresponding Authors
nicoletta.larocca@unipd.it, mariano.battistuzzi@unipd.it

Keywords: M-dwarf spectrum, Oxygenic photosynthesis, Cyanobacteria, Light acclimation, Laboratory simulations, Biosignatures

**
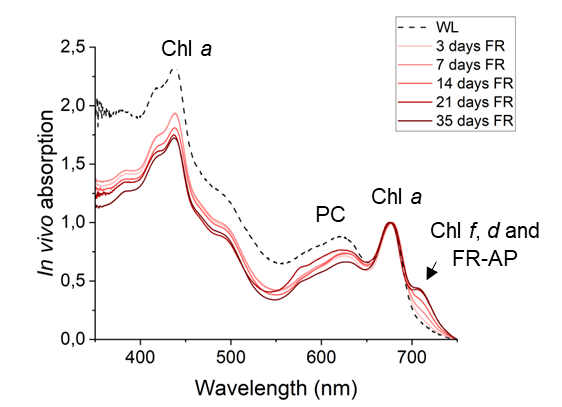
**

Figure S1. *In vivo* absorption spectra of PCC6912 at different timings of exposure to far-red light. Absorption from pigments involved in the FaRLiP response is highlighted with a black arrow. FR: Far-red light; Chl *a*: Chlorophyll *a*; PC: Phycocyanin; Chl *f*: Chlorophyll *f*; Chl *d*: Chlorophyll *d*; FR-AP: far-red induced allophycocyanin. Spectra are normalized at 680 nm (arbitrary unit) to highlight FaRLiP.

**
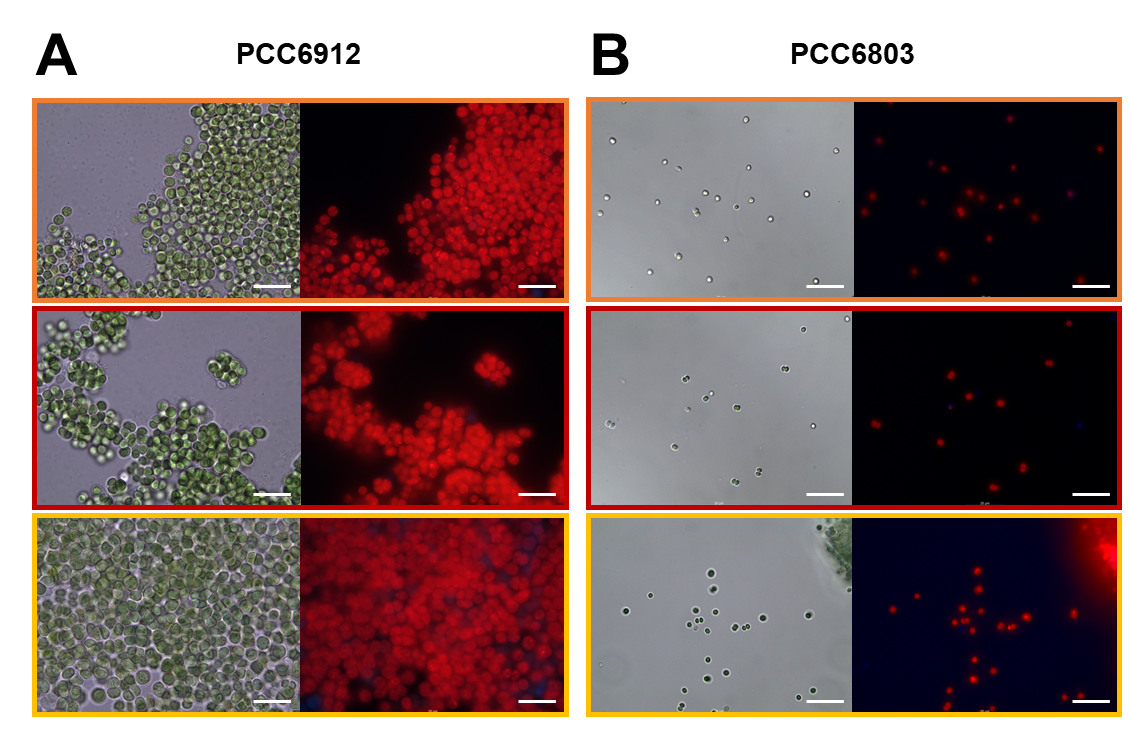
**

Figure S2. Bright-field and fluorescence optical microscopy of PCC6912 (A) and PCC6803 (B) samples after 21 days of exposure to the selected light spectra. Images were obtained through an optical microscope (Leica DM6B, Leica, Wetzlar, Germany), utilizing white and fluorescence lights, maintaining the acquisition settings for each sample. Fluorescence images highlight the vitality of cells. Red fluorescence is due to chlorophylls and is an indicator of the viability of the cells; blue fluorescence highlights dead cells. Orange: M7; red: FR; yellow: SOL. Scale bars: 20 µm.


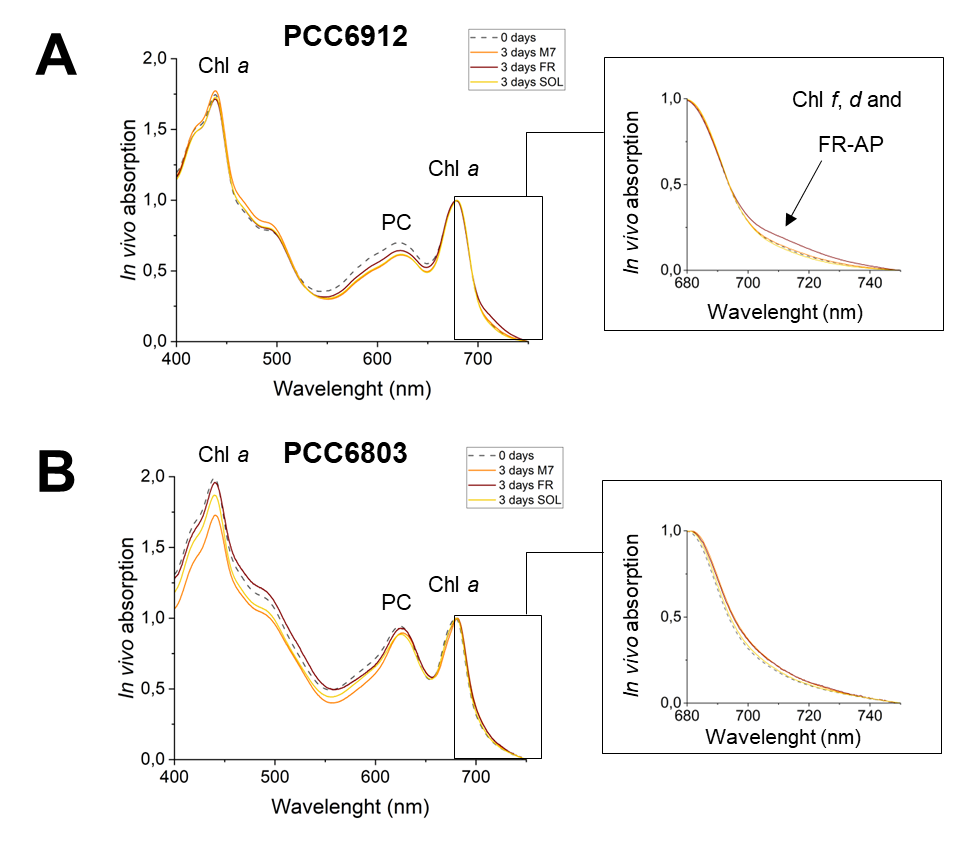


Figure S3. *In vivo* absorption spectra of PCC6912 (A) and PCC6803 (B) after 3 days of exposure. For each strain in the right box is reported the zoom of the 680-750 nm spectrum region. Where present, absorption from pigments involved in the FaRLiP response is highlighted with a black arrow. M7: M-dwarf light; FR: Far-red light; SOL: Solar light; Chl *a*: Chlorophyll *a*; PC: Phycocyanin; Chl *f*: Chlorophyll *f*; Chl *d*: Chlorophyll *d*; FR-AP: far-red induced allophycocyanin. Spectra are normalized at 680 nm (arbitrary unit) to highlight FaRLiP.

**
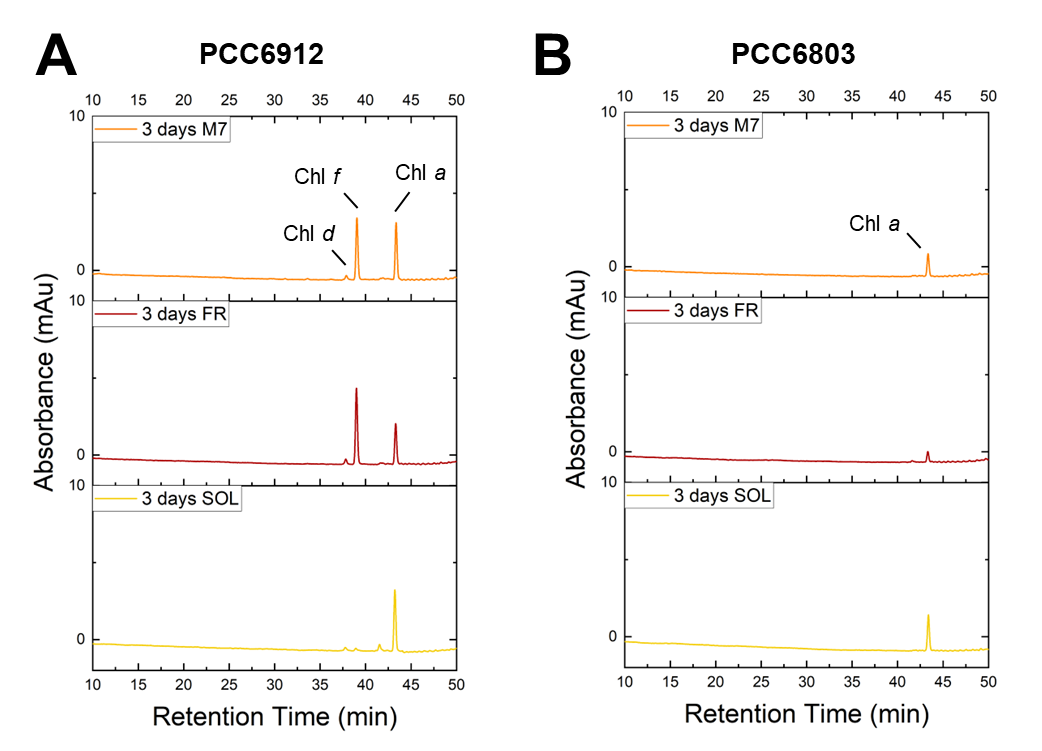
**

Figure S4. HPLC Chromatogram at 705 nm of PCC6912 (A) and PCC6803 (B) samples after 3 days of exposure. M7: M-dwarf light; FR: Far-red light; SOL: Solar light; Chl *a*: Chlorophyll *a*; Chl *f*: Chlorophyll *f*; Chl *d*: Chlorophyll *d*;

Table S1. LEDs utilized in the Solar light simulator (SOL).


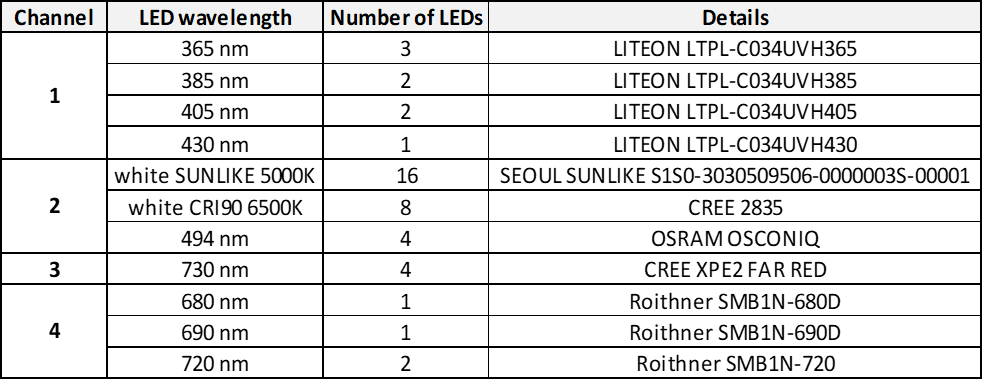


Table S2. CO_2_ and O_2_ sensors utilized in the Atmosphere Simulator Chamber.
